# Rethinking Alzheimer's Disease Therapy: From Amyloid‐Centric Approaches to Multi‐Target Phytochemical Strategies

**DOI:** 10.1111/jnc.70506

**Published:** 2026-07-14

**Authors:** Emre Aktaş, İlay Yurt, Yağmur Nisa Cerlet, Haşmet Ayhan Hanağası

**Affiliations:** ^1^ Faculty of Art and Science, Molecular Biology and Genetics Yıldız Technical University Istanbul Türkiye; ^2^ Behavioral Neurology and Movement Disorders Unit, Department of Neurology, Istanbul Faculty of Medicine Istanbul University Istanbul Turkey

**Keywords:** Alzheimer's disease, amyloid‐β, disease‐modifying strategies, multi‐target therapy, neuroinflammation, oxidative stress, phytochemicals, tau pathology

## Abstract

Alzheimer's disease (AD) is the leading cause of dementia, characterized by irreversible neuronal loss and progressive cognitive decline. The disease is driven by complex and interconnected pathological processes, including amyloid‐β plaque deposition and tau neurofibrillary tangle formation, which converge on neuroinflammation, oxidative stress, synaptic dysfunction, and widespread neuronal network failure. Although recently approved antibody‐based therapies such as lecanemab and donanemab effectively reduce cerebral amyloid burden, their clinical benefits remain modest and are accompanied by significant safety concerns, including amyloid‐related imaging abnormalities (ARIA). Current pharmacological strategies predominantly rely on single‐target mechanisms, an approach increasingly recognized as insufficient to address the multifactorial neurobiology of AD. This review critically evaluates the limitations of conventional amyloid‐centric therapeutic strategies and contrasts them with emerging multi‐target approaches based on phytochemicals. We synthesize current experimental and translational evidence to present a mechanistic framework illustrating how plant‐derived bioactive compounds, including flavonoids and polyphenols, function as systems‐level modulators of AD pathology rather than purely symptomatic agents. Particular emphasis is placed on their coordinated actions on amyloid processing via BACE1 inhibition, restoration of tau homeostasis through GSK‐3β/PP2A regulation, attenuation of neuroinflammatory signaling, enhancement of endogenous antioxidant defenses through Nrf2 activation, and preservation of synaptic integrity. When considered collectively, the available evidence supports the concept that multi‐target phytochemical strategies represent a biologically congruent and neurologically relevant paradigm for Alzheimer's disease therapy. Future progress will likely depend on integrating these compounds into broader polypharmacological and multidomain intervention frameworks, together with lifestyle‐based strategies, repurposed drugs, anti‐amyloid therapies, and rigorous translational validation.

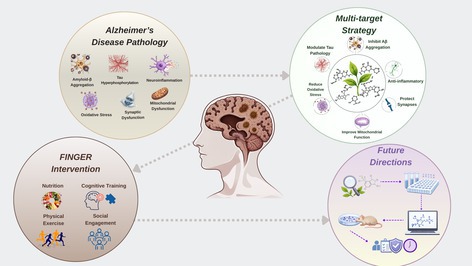

## Graphical abstract

Alzheimer's disease is driven by interconnected pathological processes, including amyloid‐β aggregation, tau hyperphosphorylation, neuroinflammation, oxidative stress, synaptic dysfunction, and mitochondrial impairment. While recently approved anti‐amyloid therapies represent an important advance, their modest clinical benefits highlight the limitations of single‐target approaches. This review proposes that multi‐target phytochemical strategies—plant‐derived compounds capable of simultaneously modulating multiple disease pathways—combined with FINGER‐type multidomain lifestyle interventions, offer a more biologically congruent framework for Alzheimer's disease therapy. Future directions include in silico screening, in vitro and in vivo validation, and translation to clinical applications.

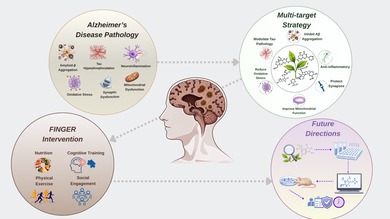

**Abbreviations**
AChE: acetylcholinesterase
AD: Alzheimer's disease
APOE: apolipoprotein E
APP: amyloid precursor protein
ARIA: amyloid‐related imaging abnormalities
Aβ: amyloid‐beta
BACE1: beta‐site APP‐cleaving enzyme 1
BBB: blood–brain barrier
FDA: Food and Drug Administration
GSK‐3β: glycogen synthase kinase‐3 beta
HO‐1: heme oxygenase‐1
IL‐1β: interleukin‐1 beta
IL‐6: interleukin‐6
LTD: long‐term depression
LTP: long‐term potentiation
NFTs: neurofibrillary tangles
NF‐κB: nuclear factor kappa B
NMDA: N‐Methyl‐D‐aspartate
Nrf2: nuclear factor erythroid 2‐related factor 2
PP2A: protein phosphatase 2A
ROS: reactive oxygen species
SOD: superoxide dismutase
TNF‐α: tumor necrosis factor alpha

## 1 Introduction

**FIGURE 1:**
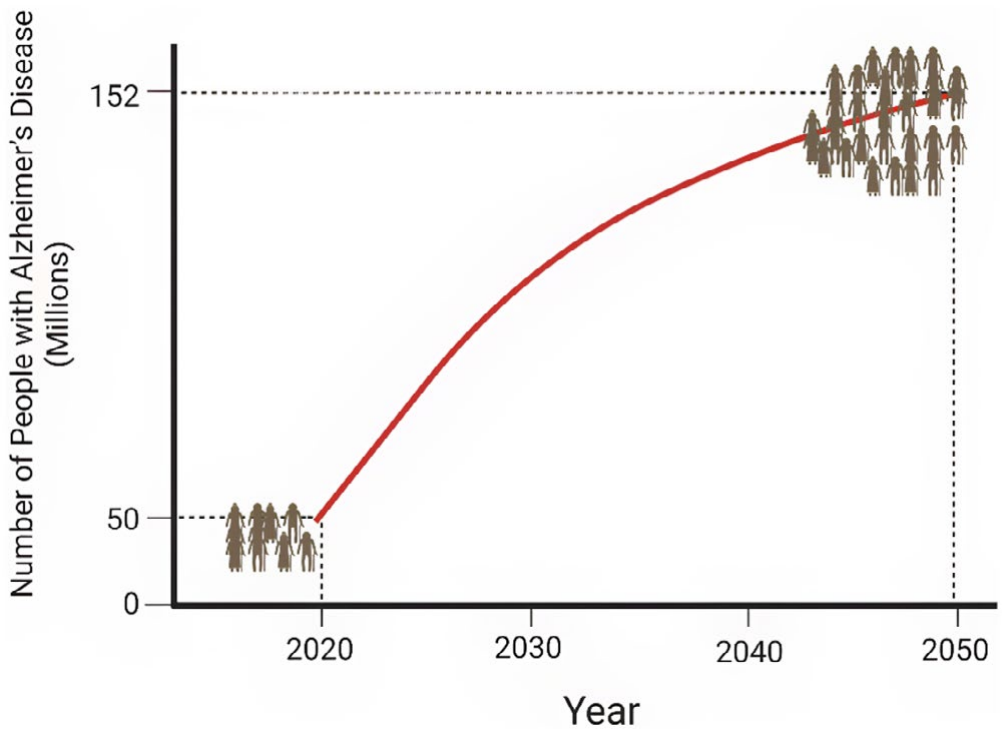
Global prevalence of Alzheimer's disease was estimated at approximately 50 million cases in 2020 and is projected to increase to around 152 million by 2050 (He et al. 2025).

Alzheimer's disease is a multifactorial neurodegenerative disorder driven by the interplay of aging, oxidative stress, genetic susceptibility and systemic metabolic and vascular disturbances (Singh et al. 2024). As the global prevalence of neurodegenerative disorders continues to rise, AD has emerged as the most common and devastating condition, representing the leading cause of dementia and one of the most formidable public health challenges of the 21st century (Scheltens et al. 2021; Job et al. 2023; Heneka et al. 2025). Characterized by irreversible and progressive neuropathological processes, AD accounts for approximately 70% of all dementia cases and currently affects nearly 50 million individuals worldwide, with global prevalence expected to approach 152 million by 2050 (He et al. 2025) (Figure 1). This projected increase is expected to impose a profound emotional, social, and economic burden (Breijyeh and Karaman 2020) with global dementia‐related costs already exceeding one trillion US dollars annually (Gustavsson et al. 2023). Despite this escalating global impact, available pharmacological therapies remain limited in efficacy. While several approved pharmacological agents provide symptomatic relief, none are capable of halting or reversing the underlying neurodegenerative progression (Breijyeh and Karaman 2020). Consequently, elucidating the etiology, pathological progression, and potential therapeutic strategies of AD remains a major focus of contemporary neuroscience and biomedical research (Alkhalifa et al. 2025). Importantly, AD‐related neuropathological changes can be detected in vivo through amyloid‐β and tau biomarkers during a prolonged preclinical phase that may persist for many years in the absence of overt clinical symptoms (Gustavsson et al. 2023). While some individuals remain in this asymptomatic stage throughout their lifetime, a substantial proportion develop measurable cognitive decline consistent with mild cognitive impairment. In individuals with positive biomarkers indicative of Alzheimer's pathology, this stage is referred to as prodromal AD. On average, the prodromal phase lasts approximately 3–5 years and represents a critical transitional period, after which many—but not all—individuals progress to AD dementia (Gustavsson et al. 2023). Population‐based studies further indicate that among individuals aged 70 years and older, a considerable proportion exhibit AD‐related neuropathological changes, with distinct distributions across preclinical, prodromal, and dementia stages (Aarsland et al. 2025).

For several decades, the prevailing conceptual framework guiding therapeutic development has been the amyloid cascade hypothesis, which positions amyloid‐β (Aβ) accumulation as the primary initiating event in AD pathogenesis (Alkhalifa et al. 2025). This paradigm has driven a shift from purely symptomatic interventions—such as acetylcholinesterase inhibitors and NMDA receptor antagonists—toward disease‐modifying strategies aimed at reducing cerebral amyloid burden (Zhang et al. 2025). More recently, monoclonal antibodies including lecanemab and donanemab have demonstrated the ability to markedly lower amyloid deposition within the brain (Zhang et al. 2025; Avgerinos et al. 2024). However, these biological effects have not translated into the transformative clinical benefits once anticipated (Zhang et al. 2025; Avgerinos et al. 2024). Symptomatic therapies are often constrained by dose‐limiting adverse effects, whereas anti‐amyloid antibodies provide only modest cognitive benefit and are associated with substantial safety concerns, most notably amyloid‐related imaging abnormalities. In addition, the high financial cost and logistical complexity of these biologic therapies, including the requirement for intensive magnetic resonance imaging surveillance, raise critical concerns regarding long‐term clinical feasibility and healthcare sustainability (Alkhalifa et al. 2025; Wang et al. 2025).

Collectively, the limited efficacy, safety liabilities, and economic burden of current amyloid‐centric approaches have prompted renewed interest in alternative therapeutic paradigms that more effectively address the multifactorial nature of Alzheimer's disease (Jurcău et al. 2022; Buccellato et al. 2021). It is now widely recognized that AD pathogenesis extends beyond protein aggregation alone and encompasses a complex interplay of oxidative stress, chronic neuroinflammation, mitochondrial dysfunction, synaptic failure, and blood–brain barrier disruption (Jurcău et al. 2022; Buccellato et al. 2021). Within this broader pathogenic framework, increasing attention has been directed toward network‐based pharmacological strategies and the therapeutic potential of phytochemicals (Sharifi‐Rad et al. 2022). Unlike conventional single‐target synthetic agents, plant‐derived bioactive compounds often exhibit pleiotropic properties, enabling simultaneous modulation of multiple disease‐relevant pathways, including antioxidant defense, inflammatory signaling, and protein aggregation (Sharifi‐Rad et al. 2022; Kim et al. 2024). In this review, we explore the neuropathological complexity of Alzheimer's disease, critically assess the limitations of current amyloid‐focused therapies, and examine the mechanistic rationale for phytochemicals as multi‐target neuroprotective agents capable of addressing the current therapeutic gap. Unlike conventional reviews that focus on individual pathological mechanisms, this study adopts a systems‐level approach to Alzheimer's disease, emphasizing the interconnected nature of its underlying biology and the necessity of multi‐target therapeutic strategies.

## 2 Neuropathological Hallmarks of Alzheimer's Disease

**FIGURE 2:**
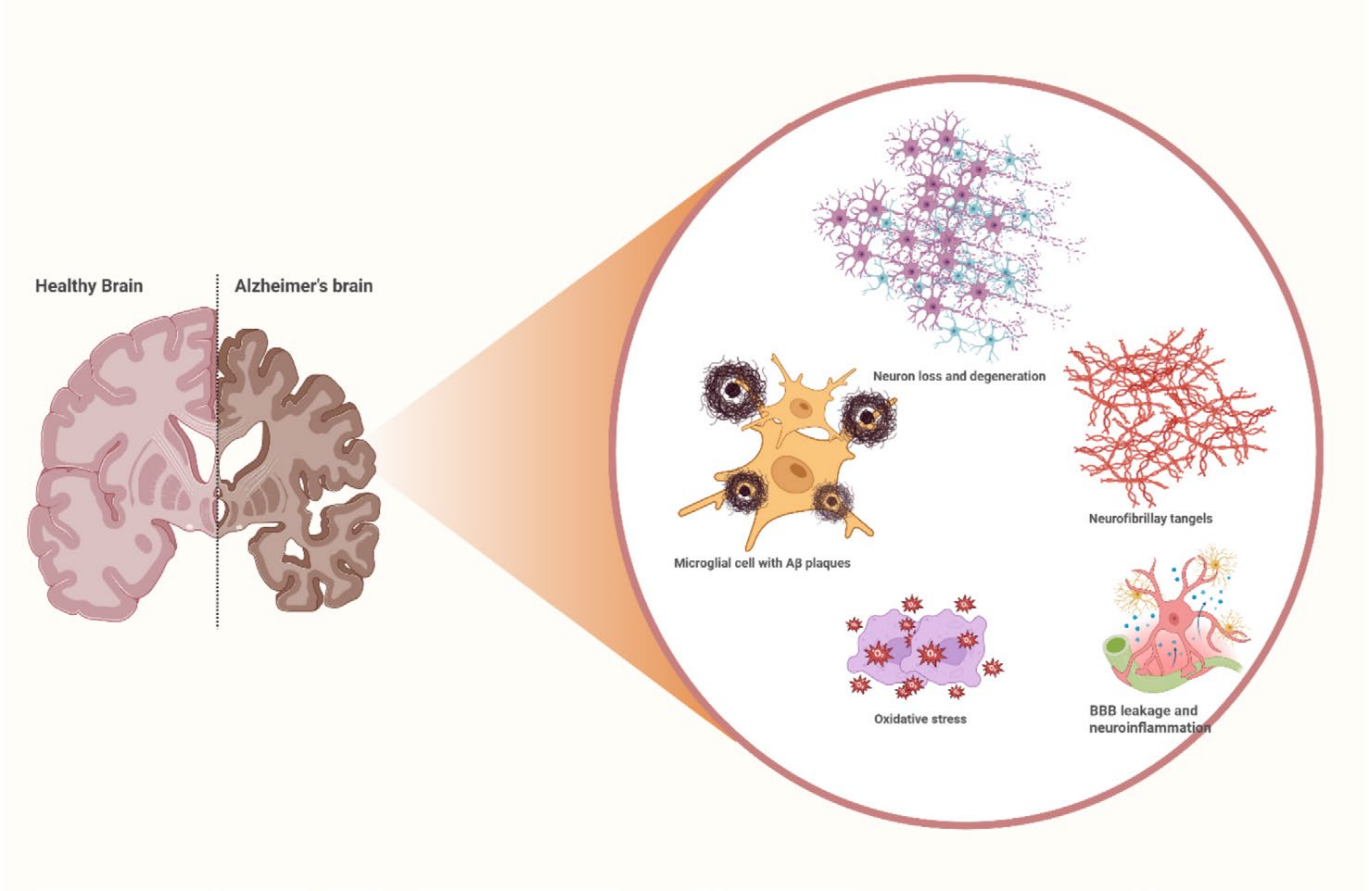
Schematic illustration comparing a healthy brain and an Alzheimer's disease brain, highlighting key pathological features associated with Alzheimer's. These include neuronal loss and degeneration, neurofibrillary tangles, amyloid‐β plaque deposition in microglial cells, oxidative stress, and blood–brain barrier (BBB) leakage accompanied by neuroinflammation.

Alzheimer's disease is clinically characterized by progressive cognitive decline and neurodegeneration yet it is now biologically defined by a distinct neuropathological profile (Jack et al. 2018). This profile includes two major hallmark lesions. The first is the extracellular accumulation of amyloid‐β peptides which form diffuse and neuritic plaques within the brain parenchyma and cerebral vasculature. The second is the intraneuronal deposition of hyperphosphorylated tau protein which aggregates into neurofibrillary tangles (NFTs) and neuropil threads within damaged neuronal processes (Breijyeh and Karaman 2020; Long and Holtzman 2019; Wójcik et al. 2024) (Figure 2). Aβ pathology disrupts neuronal communication and promotes oxidative stress and chronic neuroinflammation, with soluble Aβ oligomers identified as particularly neurotoxic species that impair synaptic function (Pulido et al. 2020). Tau pathology develops when tau becomes abnormally hyperphosphorylated and detaches from microtubules leading to its misfolding and aggregation into NFTs. Increasing evidence shows that tau pathology correlates more strongly than Aβ with both ongoing and long‐term neurodegeneration and with the extent of cognitive impairment observed in patients with AD (Svenningsson et al. 2024). As the disease advances, AD presents two broad categories of neuropathological change. Positive lesions consist of accumulated structures such as Aβ plaques, NFTs, neuropil threads and dystrophic neurites. Negative lesions refer to progressive neuronal, synaptic and neuropil loss that contributes to widespread brain atrophy (Breijyeh and Karaman 2020). In addition to Aβ and tau, the activation of glial cells has been shown to influence numerous molecular and cellular pathways that contribute to AD pathogenesis (Guo et al. 2020). Taken together, these neuropathological mechanisms reveal a complex and interconnected disease process in which Aβ accumulation, tau aggregation and glial activation reinforce one another. This interplay shapes the molecular and structural landscape of AD and drives its progressive clinical manifestation.

### 2.1 Amyloid‐Beta Plaques (Aβ Plaques)

Amyloid‐beta is generated from amyloid precursor protein (APP) through sequential cleavage by β‐secretase (BACE1) and γ‐secretase, producing Aβ40 and the more hydrophobic and aggregation‐prone Aβ42 isoform (Sadigh‐Eteghad et al. 2015; Vogt et al. 2023). These peptides undergo a stepwise aggregation process in which monomers first assemble into soluble oligomers, then into protofibrils, and finally into mature fibrils and plaques. Among these species, soluble oligomers are considered the most neurotoxic, as they disrupt synaptic function both extracellularly and intracellularly (Vogt et al. 2023; Niu et al. 2024). Aβ also promotes tau pathology (Zhang et al. 2023). Oligomeric Aβ activates kinases driving tau hyperphosphorylation, its detachment from microtubules, and the formation of neurofibrillary tangles (Guo et al. 2020; Zhang et al. 2023). This creates a pathogenic feedback loop in which Aβ‐induced tau changes amplify synaptic failure and neuronal loss (Guo et al. 2020; Zhang et al. 2023). Moreover, Aβ accumulation activates microglia and astrocytes, leading to chronic neuroinflammation. Although initially involved in Aβ clearance, glial cells gradually lose phagocytic efficiency, which further accelerates peptide aggregation and neuronal damage (Guo et al. 2020). Together, these mechanisms explain why Aβ‐directed therapies aim to reduce monomer and oligomer production, neutralize oligomeric species, and disrupt Aβ–tau interactions. Such strategies are currently being evaluated in clinical studies and show promise in slowing disease progression (Zhang et al. 2023).

### 2.2 Neurofibrillary Tangles

Tau is a neuronal microtubule‐associated protein that is highly expressed under physiological conditions and is fundamental for microtubule assembly, stabilization and the maintenance of axonal architecture (Ge et al. 2025). By regulating axonal growth, neuronal polarity, intracellular cargo trafficking and synaptic plasticity, tau serves as an essential structural and functional scaffold required for preserving neuronal connectivity (Chen and Yu 2023; Sinsky et al. 2021). In Alzheimer's disease, tau undergoes pathological hyperphosphorylation, loses its affinity for microtubules and adopts misfolded conformations that aggregate into insoluble neurofibrillary tangles (Chen and Yu 2023; Rawat et al. 2022). NFT burden shows a robust correlation with both the severity of cognitive decline and the extent of neurodegeneration in AD (Chen and Yu 2023). Emerging evidence further indicates that smaller, soluble tau oligomers—often referred to as the “tau we cannot see”—exert pronounced synaptotoxic effects and may precede and potentiate fibrillar tau deposition (Chen and Yu 2023). Cumulatively, these insights highlight tau dysregulation as a central pathological axis in AD. Therapeutic strategies aimed at reducing tau hyperphosphorylation, modulating tau kinases or inhibiting tau fibrillization represent promising avenues, and tau‐targeted interventions continue to show potential for altering the trajectory of disease progression (Liu et al. 2022; Wang et al. 2023).

### 2.3 Neuroinflammation

Neuroinflammation is increasingly recognized as a core pathological axis in AD, acting not only as a downstream response but as a potent amplifier of both amyloid‐β and tau pathology (Onyango et al. 2021; Roveta et al. 2024). Under physiological conditions, microglia and astrocytes maintain synaptic homeostasis, regulate neurotransmitter balance and support blood–brain barrier (BBB) integrity (Si et al. 2023). However, exposure to Aβ plaques and tau aggregates drives these glial cells into a state of sustained activation, shifting them toward a chronically pro‐inflammatory phenotype characterized by increased release of cytokines, chemokines and neurotoxic mediators (Thakur et al. 2023). This persistent activation profoundly impairs microglial Aβ‐clearance capacity, thereby accelerating extracellular Aβ accumulation and potentiating neuronal injury (Onyango et al. 2021). Moreover, microglial activation has been shown to contribute substantially to tau propagation; in postmortem AD brain tissue, the proportion of activated microglia closely mirrors regional tau accumulation, and mechanistic studies suggest that microglia act upstream to promote tau hyperphosphorylation and subsequent cognitive impairment (Chen and Yu 2023). The resulting inflammatory milieu disrupts BBB integrity, enabling the infiltration of peripheral immune cells into the central nervous system and further escalating neuroinflammatory burden (Roveta et al. 2024). Inflammatory and oxidative stress‐driven pathways also compromise oligodendrocyte function, reduce key myelin proteins and impair neuronal communication (Onyango et al. 2021). Crucially, neuroinflammation arises at the earliest stages of AD, often preceding clinical symptoms, and is believed to act as a mechanistic bridge linking chronic Aβ accumulation to the propagation of tau pathology (Roveta et al. 2024; Si et al. 2023). Together, these observations establish neuroinflammation not as a secondary by‐product but as a primary pathogenic driver that shapes disease onset, accelerates progression and exacerbates neuronal degeneration.

### 2.4 Synaptic Dysfunction

Synaptic dysfunction is now regarded as one of the earliest and most defining pathological processes in Alzheimer's disease, emerging prior to overt amyloid or tau deposition and demonstrating the strongest correlation with cognitive decline (Yu, Chen, et al. 2024; Wu et al. 2021). Extensive evidence indicates that synapse loss is the most reliable biological predictor of cognitive impairment, with reductions in synaptic density closely linked to deficits in both immediate and delayed recall (Pei et al. 2020; Meftah and Gan 2023). Ultrastructural electron microscopy studies further reveal that individuals with mild AD exhibit synaptic reductions reaching up to 55% compared with cognitively normal controls (Pei et al. 2020). Early synaptic pathology is particularly pronounced within dendritic compartments, with layers II and III of the frontal and parietal cortex showing substantial vulnerability (John and Reddy 2021). As AD progresses, elevated amyloid‐β burden disrupts synaptic plasticity, alters synaptic vesicle trafficking, and induces morphological deterioration at synaptic sites (John and Reddy 2021; Zhang et al. 2022). Aβ‐mediated impairments in synaptic transmission—including attenuation of long‐term potentiation (LTP)—have been consistently demonstrated in transgenic mouse models (Zhang et al. 2022). Aβ and hyperphosphorylated tau synergistically amplify synaptic damage (Wu et al. 2021; Zhang et al. 2022). Soluble tau oligomers, which are more synaptotoxic and appear earlier than mature neurofibrillary tangles, are capable of inducing marked synapse loss even in the absence of global neuronal degeneration (Wu et al. 2021; Zhang et al. 2022). These pathological species converge on shared mechanisms involving NMDA receptor dysregulation, increased reactive oxygen species (ROS) production, and oxidative stress, collectively accelerating the functional collapse of synaptic networks (Zhang et al. 2022). Glial dysregulation further shapes synaptic vulnerability in AD. Microglia and astrocytes—normally essential for synapse maintenance and neuronal development—adopt maladaptive phenotypes that drive aberrant synaptic pruning and disrupt neuron–glia communication (Yu, Chen, et al. 2024). Such disruptions lead to progressive disassembly of synaptic circuits, weakened connectivity, and deficits in learning and memory (Yu, Chen, et al. 2024; Meftah and Gan 2023). As a whole, these findings establish AD fundamentally as a synaptopathy, in which early and progressive synaptic failure dictates the trajectory of neurodegeneration and cognitive decline (Meftah and Gan 2023).

### 2.5 Oxidative Stress

Oxidative stress is widely recognized as a central pathogenic mechanism in Alzheimer's disease, integrating multiple hypotheses including amyloid, tau, inflammation, metal‐ion imbalance and mitochondrial dysfunction (Bai et al. 2022; Dhapola et al. 2024). For decades, elevated oxidative damage has been consistently observed in atrophied AD brain tissue, underscoring its fundamental role in disease progression (Cassidy et al. 2020). Mitochondrial dysfunction is both a major source and target of oxidative injury. Impaired electron transport increases reactive oxygen species, which in turn amplify Aβ and tau pathology, creating a self‐perpetuating toxic cycle (Dhapola et al. 2024). ROS induce lipid, protein, and nucleic‐acid peroxidation, leading to neuronal death and disruption of intracellular signaling pathways (Cassidy et al. 2020). High levels of oxidative markers at synapses further demonstrate that oxidative stress directly compromises synaptic integrity (Dhapola et al. 2024). Neurons are especially vulnerable due to their high metabolic demand and abundance of polyunsaturated fatty acids, which are readily oxidized (Valverde‐Salazar et al. 2023). Reduced glutathione levels during aging exacerbate susceptibility to oxidative injury, while ROS accumulation promotes tau hyperphosphorylation and Aβ‐mediated mitochondrial membrane damage (Valverde‐Salazar et al. 2023). Mitochondrial dysfunction is considered one of the earliest events in AD pathogenesis, emphasizing its central role (Valverde‐Salazar et al. 2023). Oxidative stress acts through macromolecular peroxidation, Aβ–metal redox reactions and mitochondrial impairment, all of which accelerate Aβ and phosphorylated tau accumulation (Cassidy et al. 2020). It also contributes to stress granule formation and neuronal degeneration, particularly when antioxidant defenses are diminished (Dhapola et al. 2024). Overall, these findings define oxidative stress as a primary pathogenic driver in AD and a critical target for therapeutic intervention (Bai et al. 2022).

## 3 Etiological and Risk Factors in Alzheimer's Disease

Alzheimer's disease is a multifactorial neurodegenerative disorder driven by the interplay of aging, oxidative stress, genetic susceptibility and systemic metabolic and vascular disturbances (Singh et al. 2024). Aging is a major upstream factor that elevates reactive oxygen species and activates oxidative stress–dependent cascades leading to neuronal loss (Magalingam et al. 2018). This oxidative environment accelerates tau hyperphosphorylation and aggregation, positioning tau dysregulation as a key determinant of disease progression (Ge et al. 2025). Although no single mutation explains sporadic AD, genetic background strongly shapes vulnerability. The APOE ε4 allele is the most influential common risk factor and enhances Aβ deposition, impairs microglial clearance and promotes tau phosphorylation (Rostagno 2022). Rare variants in TREM2, PLCG2, and ABI3 amplify innate immune activation, while early‐onset familial AD is primarily caused by autosomal‐dominant mutations in APP, PSEN1 and PSEN2 (Singh et al. 2024; Scarano et al. 2025). Metabolic impairments such as brain insulin resistance, mitochondrial dysfunction and reduced glucose utilization further intensify oxidative stress and accelerate neurodegeneration (Rostagno 2022). Western dietary patterns exacerbate these abnormalities, especially in APOE‐ε4 carriers (Singh et al. 2024). Defective lysosomal acidification and impaired autophagy hinder the clearance of toxic proteins, reinforcing aggregate accumulation (Singh et al. 2024; Rostagno 2022). Epidemiological evidence highlights the importance of modifiable risk factors in Alzheimer's disease. According to the Lancet Commission, effective control of hypertension, smoking and physical inactivity could prevent up to half of AD cases. Beyond these core factors, the literature highlights a broad range of modifiable risks, including sensory impairments, depression, metabolic and vascular conditions, unhealthy lifestyle behaviors, social isolation, traumatic brain injury, and environmental exposures such as air pollution. In parallel, several non‐pharmacological approaches, including physical activity, cognitive stimulation, and reduced social isolation, have been proposed to alleviate Alzheimer's disease–associated symptoms (Scarano et al. 2025). Substantial pathophysiological and clinical evidence links obesity and obesity‐related comorbidities, including insulin resistance, hyperglycaemia and type 2 diabetes, with an increased risk of Alzheimer's disease (Alford et al. 2018). Collectively, these findings support a model in which genetic predisposition defines baseline susceptibility, while metabolic, vascular and lifestyle‐related stressors critically influence the onset and trajectory of Alzheimer's disease.

## 4 Limitations of Current Pharmacological Therapies

Since the early development of therapeutic strategies for Alzheimer's disease, active and passive immunotherapies targeting amyloid‐β have demonstrated the potential to modify Aβ deposition and have therefore been extensively investigated in animal models and clinical settings (Buccellato et al. 2023). While the majority of these approaches remain under clinical evaluation, a limited number of agents have achieved regulatory approval (Buccellato et al. 2023). To date, FDA‐approved treatments for AD include symptomatic therapies such as acetylcholinesterase inhibitors and NMDA receptor antagonists, as well as a small number of anti‐amyloid monoclonal antibodies developed as disease‐modifying therapies for early‐stage AD (Table 1). As of the index date of January 1, 2025, the Alzheimer's disease clinical trial pipeline comprised 182 ongoing studies evaluating 138 therapeutic agents. This included 48 trials assessing 31 drugs in Phase 3, 86 trials examining 75 agents in Phase 2, and 48 trials evaluating 45 drugs in Phase 1. Additionally, 16 of these trials were long‐term extension studies of agents previously tested in earlier phases (Cummings, Zhou, et al. 2025). Given their potential to influence disease progression and their extensive representation in the literature, lecanemab and donanemab are used as reference frameworks for a more detailed examination of current therapeutic strategies for Alzheimer's disease.

**TABLE 1:** Overview of FDA‐approved pharmacological treatments for Alzheimer's disease, including symptomatic and disease‐modifying therapies.

Medication	Mechanism of action	Approved indication (FDA)	Approved
Lecanemab	Targets amyloid‐β protofibrils and plaques (Swanson et al. 2021).	Disease‐modifying therapy for early AD	Yes
Donanemab	Specific for pyroglutamate‐modified amyloid‐β species (Buccellato et al. 2023).	Disease‐modifying therapy for early AD	Yes
Donepezil	Acetylcholinesterase inhibitor; enhances cholinergic transmission (Varadharajan et al. 2023; Adlimoghaddam et al. 2018).	Alzheimer's disease (symptomatic therapy)	Yes
Rivastigmine	Reversible cholinesterase inhibitor; inhibits both acetylcholinesterase and butyrylcholinesterase (Patel and Gupta 2025).	Mild‐to‐moderate dementia of the AD	Yes
Galantamine	Reversible competitive inhibitor of AChE; potentiates the action of ACh on nicotinic receptors (Varadharajan et al. 2023).	Mild‐to‐moderate AD	Yes
Memantine	NMDA receptor‐antagonist; blocks current flow through glutamatergic NMDA receptors and inhibits calcium influx (Varadharajan et al. 2023; Johnson and Kotermanski 2006).	Moderate‐to‐severe AD	Yes

### 4.1 Lecanemab

Lecanemab (Leqembi TM) is a humanized monoclonal antibody that selectively targets soluble amyloid‐β protofibrils, which are considered among the most neurotoxic intermediates in the amyloid aggregation cascade (van Dyck et al. 2023). By preferentially binding protofibrillar Aβ rather than monomeric or insoluble fibrillar forms, lecanemab is designed to neutralize pathogenic amyloid assemblies implicated in early synaptic dysfunction and cognitive decline (van Dyck et al. 2023).

### 4.2 Donanemab

Donanemab (donanemab‐azbt; Kisunla) is a humanized amyloid‐β–directed monoclonal antibody developed for the treatment of early symptomatic Alzheimer's disease, including mild cognitive impairment and mild dementia (Kang 2024; Dodel and Frölich 2025). Unlike antibodies targeting soluble amyloid species, donanemab selectively binds an N‐terminally truncated pyroglutamate form of amyloid‐β (pGlu3‐Aβ), which is predominantly associated with cerebral amyloid plaques, particularly cored plaques within the central nervous system [55, 56]. Preclinical and clinical studies indicate that donanemab exhibits strong plaque‐binding activity and effectively reduces cerebral amyloid deposition; however, whether this amyloid removal translates into sustained clinical benefit remains unclear, underscoring ongoing uncertainty regarding its therapeutic impact in Alzheimer's disease (Dodel and Frölich 2025; Rashad et al. 2022).

### 4.3 Clinical Efficacy and Limitations

Clinical trials have demonstrated dose‐ and time‐dependent reductions in brain amyloid burden with lecanemab treatment, accompanied by a statistically significant slowing of clinical deterioration over an 18‐month period compared with placebo (van Dyck et al. 2023). Nevertheless, the magnitude of this effect remains modest. Accumulating evidence suggests that reductions in amyloid pathology do not consistently correspond to improvements that meet established thresholds for clinical relevance, underscoring important limitations in the therapeutic impact of lecanemab despite its measurable biological effects (Wright et al. 2023). Similarly, clinical trials indicate that donanemab treatment is associated with substantial amyloid reduction and preliminary evidence of delayed cognitive and functional decline in patients with mild‐to‐moderate Alzheimer's disease (Rashad et al. 2022).

### 4.4 Safety Profile and Treatment Burden

Safety concerns represent a major limitation of current anti‐amyloid immunotherapies. Lecanemab treatment has been associated with substantial adverse effects, most notably amyloid‐related imaging abnormalities such as cerebral edema and intracerebral hemorrhage, alongside unresolved long‐term risks and relatively high treatment costs, all of which complicate its overall risk–benefit profile (Espay et al. 2024; Angelova et al. 2024; Kong et al. 2025). Similarly, donanemab therapy has shown a consistently elevated risk of ARIA compared with placebo, with incidence rates ranging from 26.1% to 30.5% across clinical trials and open‐label extensions (Rashad et al. 2022; Zimmer et al. 2025). Although ARIA‐E events are frequently transient and asymptomatic, ARIA can be severe, life‐threatening, or fatal, necessitating rigorous safety monitoring during treatment (Zimmer et al. 2025). Rare but serious complications, including seizures, status epilepticus, and fatal intracerebral hemorrhage, have been reported, particularly in patients presenting focal neurological symptoms or receiving thrombolytic therapy (Dodel and Frölich 2025). In addition, infusion‐related reactions occur more frequently in donanemab‐treated patients than in placebo‐treated individuals, often during early infusions (Dodel and Frölich 2025; Rashad et al. 2022). Comparative analyses further indicate that high‐clearance anti‐amyloid antibodies, including donanemab and lecanemab, exhibit lower tolerability and significantly higher risks of treatment discontinuation and ARIA than placebo (Jeremic et al. 2025). Collectively, these findings highlight that safety and tolerability concerns remain major constraints on the clinical utility of current anti‐Aβ therapies. Taken together, these findings indicate that current anti‐amyloid therapies, although biologically active, fail to adequately address the multifactorial nature of Alzheimer's disease. The modest clinical benefits observed despite substantial amyloid reduction challenge the centrality of the amyloid cascade hypothesis as a standalone therapeutic paradigm. This discrepancy suggests that targeting a single pathological axis is insufficient to halt disease progression, thereby reinforcing the need for multi‐target strategies capable of simultaneously modulating interconnected pathological processes.

## 5 Rationale for Phytochemicals as Multi‐Target Therapeutics

**FIGURE 3:**
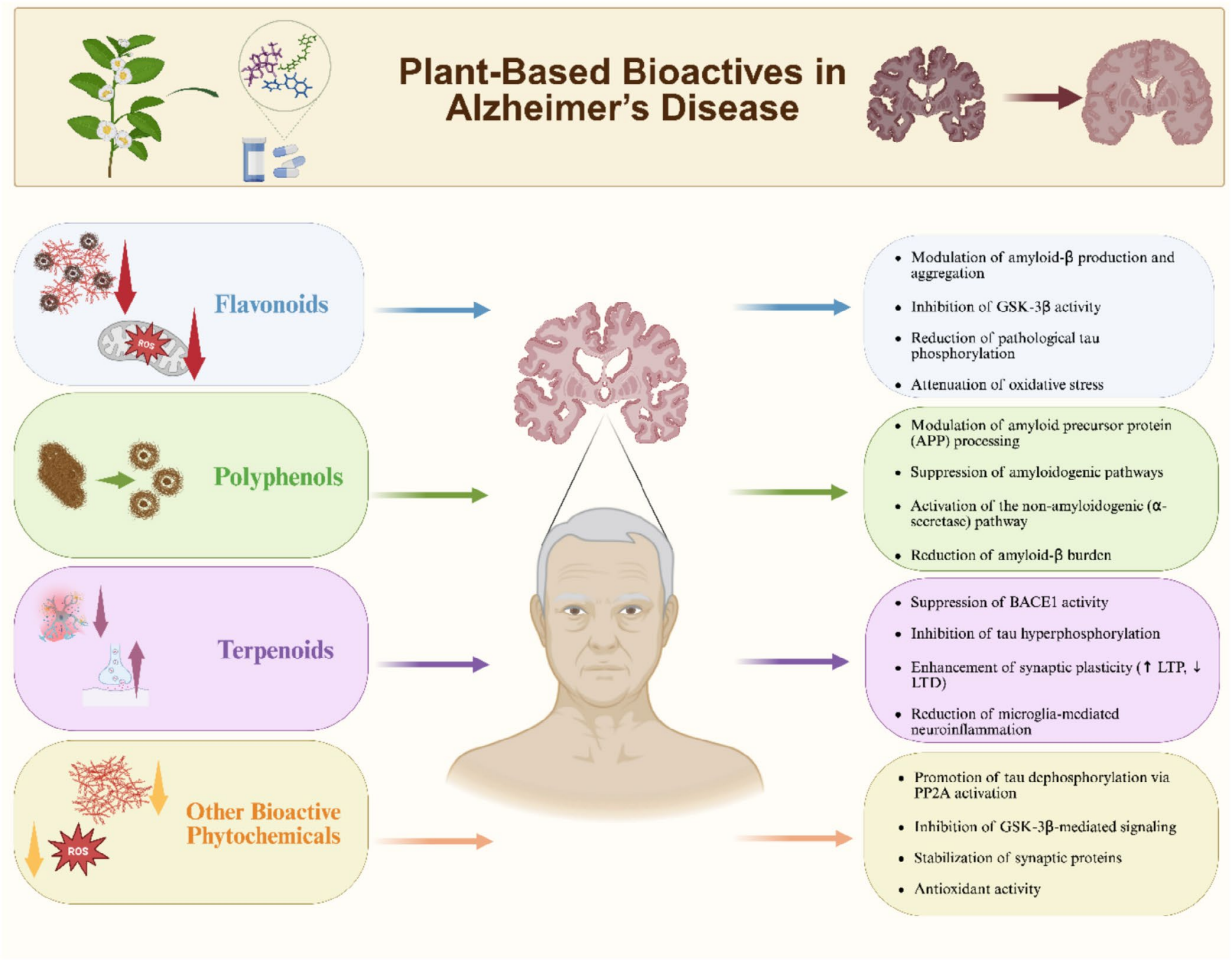
This schematic provides an overview of the major pathogenic pathways targeted by flavonoids, terpenoids, polyphenols and other phytochemicals, including modulation of amyloid precursor protein processing and amyloid‐β burden, inhibition of tau hyperphosphorylation via GSK‐3β– and PP2A‐dependent pathways, attenuation of oxidative stress and neuroinflammation, and support of synaptic plasticity and neuronal function.

Given the limited clinical efficacy and safety concerns associated with current Alzheimer's disease therapies, increasing attention has shifted toward alternative strategies capable of addressing the complex and multifactorial nature of the disease. In this context, phytochemicals—plant‐derived bioactive compounds—have attracted growing interest as potential therapeutic agents in AD research (Chihomvu et al. 2024) (Figure 3). Their growing relevance reflects the need for alternative strategies capable of modulating the complex and multifactorial pathology of AD, as well as their distinctive structural features that enable effective interactions with biological systems (Harvey et al. 2015; Lautié et al. 2020). A defining advantage of phytochemicals is their remarkable chemical diversity. In contrast to conventional synthetic libraries, which are typically constrained to a limited number of scaffolds and occupy relatively narrow regions of chemical space, phytochemicals display complex three‐dimensional architectures, high stereochemical complexity, and macrocyclic motifs shaped through evolutionary selection (Ingle et al. 2024; Rodrigues et al. 2016; Nair et al. 2020). This evolutionary refinement has optimized these molecules for interactions with biological macromolecules and contributes to their pharmacological relevance. As a result, many phytochemicals exhibit favorable biocompatibility and reduced off‐target toxicity compared with synthetic compounds, while their structural flexibility supports modulation of challenging biological targets and multi‐target activity (Chihomvu et al. 2024; Atanasov et al. 2021; Firn and Jones 2003). Recent advances in computational biology and in silico modeling have further accelerated the systematic evaluation of phytochemicals in drug discovery (Azmal et al. 2024). Within the context of neurodegenerative disorders, these compounds have demonstrated neuroprotective potential through modulation of amyloid‐β aggregation, tau pathology, and neuroinflammatory processes, supporting their consideration as candidates for next‐generation AD therapeutics (Sharifi‐Rad et al. 2022; Ghosh et al. 2025) (Figure 3).

Importantly, while phytochemicals offer a mechanistically attractive multi‐target profile, the majority of supporting evidence remains confined to in vitro and preclinical models. This represents a critical translational gap in the field. Therefore, future research must prioritize well‐designed clinical studies and pharmacokinetic optimization strategies to validate their therapeutic potential in human populations. It is important to note, however, that phytochemicals represent one category within a broader polypharmacological framework for Alzheimer's disease. The case for moving beyond single‐target approaches applies equally to combination strategies involving approved pharmacological agents (e.g., co‐administration of a cholinesterase inhibitor with an anti‐amyloid antibody), repurposed drugs targeting metabolic or vascular co‐pathologies, and structured non‐pharmacological interventions addressing modifiable risk factors such as physical inactivity, diet, sleep disturbance, and social isolation. A genuinely holistic therapeutic paradigm for AD would therefore encompass all of these co‐agent classes—with phytochemicals occupying a promising, mechanistically distinct niche—rather than treating plant‐derived compounds as the sole alternative to amyloid‐centric monotherapy. This broader perspective also raises critical questions about how trials of multi‐agent regimens should be designed and interpreted, a challenge that is discussed further in Section 7.

## 6 Phytochemicals Targeting Key AD Mechanisms

### 6.1 Anti‐Aβ Phytochemicals

The production, accumulation, and clearance of amyloid‐β peptides represent central events in the pathogenesis of Alzheimer's disease and constitute primary therapeutic targets (Liu et al. 2021). Phytochemicals can interfere with these processes by modulating the proteolytic processing of amyloid precursor protein through multiple mechanisms (Krawczuk et al. 2025).

#### 6.1.1 BACE1 Inhibition (Reduction of Aβ Production)

Given that excessive Aβ production and accumulation constitute a central pathogenic event in Alzheimer's disease, therapeutic strategies aimed at reducing Aβ generation through modulation of amyloidogenic processing have attracted considerable attention (Li et al. 2021). Genipin, a bioactive aglycone derived from *Gardenia jasminoides* , has been shown to strongly suppress the expression of beta‐site APP‐cleaving enzyme 1, the rate‐limiting enzyme in Aβ generation (Li et al. 2021). By inhibiting the amyloidogenic APP processing pathway, genipin effectively reduces the burden of toxic Aβ species (Wang et al. 2025; Li et al. 2021). 1,8‐Cineole, the major terpenoid component of eucalyptus oil, directly downregulates BACE1 activity, thereby limiting Aβ production (An et al. 2022). Dieckol, a polyphenolic compound isolated from brown algae, exerts a dual modulatory effect on APP processing (Wang et al. 2025). It downregulates the expression of both BACE1 and Presenilin‐1 (PS1), while simultaneously upregulating ADAM10 (α‐secretase), the key enzyme of the non‐amyloidogenic pathway (Yoon et al. 2021). This coordinated regulation shifts APP processing toward a neuroprotective route and provides bidirectional protection against Aβ pathology (Yoon et al. 2021).

#### 6.1.2 Anti‐Aggregation and Plaque Clearance

As discussed earlier, beyond strategies aimed at limiting Aβ production, targeting the aggregation of Aβ species and promoting the clearance of existing amyloid deposits represents a complementary therapeutic approach in Alzheimer's disease. Quercetin exhibits pronounced anti‐fibrillogenic activity by preventing the assembly of Aβ monomers into toxic oligomers and fibrils (Lou et al. 2011). Additionally, it facilitates the destabilization of pre‐formed amyloid plaques, contributing to a reduction in existing amyloid deposits (Alghamdi et al. 2022; Zamanian et al. 2025). In a similar manner, curcumin, the principal polyphenol derived from *Curcuma longa* , directly interacts with Aβ aggregates, inhibiting fibril formation and promoting the disassembly of existing plaques. Preclinical studies have demonstrated that curcumin administration leads to a significant reduction in amyloid plaque burden and mitigates Aβ‐associated neurotoxicity, highlighting its role as a multi‐targeted anti‐aggregation agent (Ege 2021; Ramalho et al. 2025). Similarly, magnolol, a neolignan isolated from the bark of *Magnolia* species, has been shown to significantly reduce Aβ(1–42) accumulation, decrease plaque burden, and protect hippocampal neurons from Aβ‐induced neurotoxicity, further supporting the therapeutic potential of anti‐aggregation strategies (Wang and Jia 2023).

### 6.2 Anti‐Tau Phytochemicals

The suppression of tau pathology is essential for preserving neuronal cytoskeletal integrity, maintaining microtubule stability, and preventing the formation of neurofibrillary tangles (Jitendra Joshi and Raja Sekhar Reddy 2024). In this context, phytochemicals primarily exert their effects through inhibition of glycogen synthase kinase‐3β (GSK‐3β) and activation of tau‐directed phosphatases, thereby restoring tau phosphorylation homeostasis (Jitendra Joshi and Raja Sekhar Reddy 2024).

#### 6.2.1 GSK‐3β Inhibition and Protection of Specific Phosphorylation Epitopes

Targeting glycogen synthase kinase‐3β, a key kinase driving pathological tau hyperphosphorylation, represents a central strategy for limiting tau‐mediated neurodegeneration in Alzheimer's disease (Jiang et al. 2016). Quercetin has been shown to activate the phosphoinositide 3‐kinase (PI3K)/Akt signaling pathway, leading to inhibitory phosphorylation of GSK‐3β at Ser9 and a consequent reduction in tau hyperphosphorylation at critical epitopes, including Ser199, Ser396, Thr205, and Thr231 (Alsaleem et al. 2024; Gong et al. 2011). In contrast, morin directly inhibits GSK‐3β with high potency, and studies in the 3xTg‐AD mouse model demonstrate that morin markedly reduces paired helical filament‐like tau accumulation and decreases tangle‐associated tau immunoreactivity (Wang et al. 2025; Kim 2021). Consistent with these findings, sulforaphane suppresses pathological tau phosphorylation at Thr205, Ser396, and Ser404 epitopes, thereby limiting neurofibrillary tangle formation (Yang et al. 2020; Zeng et al. 2010). Similarly, icariin attenuates Aβ‐induced tau hyperphosphorylation through activation of the PI3K/Akt pathway, with particularly pronounced effects at Ser396 and Ser404, highlighting its role in disrupting the pathological crosstalk between amyloid and tau signaling cascades (Yang, Fu, et al. 2023; Razgonova et al. 2019).

#### 6.2.2 Restoration of the Phosphatase/Kinase Balance: PP2A Activation

Although inhibition of pro‐inflammatory kinases such as GSK‐3β remains a primary focus, restoration of endogenous dephosphorylation mechanisms represents an equally critical axis in tau homeostasis (Li et al. 2013). In this regard, phytochemicals that can recalibrate this enzymatic equilibrium offer a more comprehensive neuroprotective strategy. Ginsenoside Rd., a triterpenoid saponin, exhibits a unique dual mechanism in the regulation of tau pathology (Shankar et al. 2008). In addition to suppressing GSK‐3β activity by reducing Tyr216 phosphorylation, it enhances the activity of Protein Phosphatase 2A (PP2A), the principal enzyme responsible for tau dephosphorylation (Li et al. 2013; Shankar et al. 2008). This coordinated modulation of kinase and phosphatase activities positions ginsenoside Rd. as a particularly promising agent for the clearance of pathological tau species and the mitigation of tau‐driven neurodegeneration (Li et al. 2013).

### 6.3 Synaptic Protection

Beyond the clearance of toxic protein aggregates, the ultimate success of any Alzheimer's disease therapy depends on its ability to preserve effective neuronal communication. Synaptic loss is widely recognized as the pathological feature most strongly correlated with cognitive decline in AD (Ebrahimi et al. 2025). Accordingly, the preservation of synaptic plasticity, particularly long‐term potentiation (LTP), together with the maintenance of synaptic structural integrity, has emerged as a critical therapeutic objective (Ebrahimi et al. 2025; Vauzour et al. 2008). In this context, accumulating evidence indicates that phytochemicals exert multifaceted neuroprotective effects by supporting synaptic function at both electrophysiological and molecular levels (Vauzour et al. 2008; Noh et al. 2013).

#### 6.3.1 Improvement of Long‐Term Potentiation (LTP)

Isoorientin has demonstrated significant protective effects on synaptic plasticity in APP/PS1 transgenic mouse models (Tan et al. 2021). Experimental studies indicate that isoorientin reverses deficits in LTP, the electrophysiological substrate of memory formation, and enhances synaptic transmission efficiency (Tan et al. 2021; Serrano et al. 2014). Parallel to these findings, Andrographolide increases the slope of field excitatory postsynaptic potentials (fEPSPs) in the hippocampal CA1 region, thereby strengthening synaptic signaling (Ji et al. 2023). In addition, it inhibits long‐term depression (LTD), contributing to the preservation of LTP and supporting synaptic adaptations underlying learning and memory (Ji et al. 2023).

#### 6.3.2 Preservation of Synaptic Proteins and Structural Integrity

While the enhancement of electrophysiological signaling is crucial, the long‐term maintenance of cognitive function necessitates the physical preservation of synaptic architecture and the stability of the neurotransmitter environment (More et al. 2012). Furthermore, phytochemicals target diverse structural and enzymatic pathways to ensure this integrity (More et al. 2012). Specifically, Oxyphylla A promotes synaptic plasticity through modulation of the Akt–GSK‐3β signaling pathway and is associated with improvements in cognitive performance (More et al. 2012). These effects are linked to enhanced stability of synaptic proteins and improved efficiency of synaptic signal transduction (More et al. 2012). Genipin exhibits a pronounced neuritogenic effect, supporting neuronal structural integrity (Liu et al. 2015). By promoting neurite outgrowth and increasing synaptic connectivity, genipin provides a structural repair and remodeling mechanism that counteracts neurodegenerative processes (Pan et al. 2019). Complementing these structural benefits, Linarin enhances synaptic function by inhibiting acetylcholinesterase (AChE), leading to increased acetylcholine levels within the synaptic cleft (Feng et al. 2015). This mechanism strengthens cholinergic neurotransmission and supports synaptic activity, addressing one of the most prominently affected neurotransmitter systems in AD (Feng et al. 2015; Sidiropoulou et al. 2023).

### 6.4 Anti‐Inflammatory & Antioxidant Effects

Neuroinflammation and oxidative stress act synergistically with GSK‐3β hyperactivity to form a pathological vicious cycle that accelerates neuronal dysfunction and cell death in Alzheimer's disease (Bian et al. 2021). Targeting these interconnected processes is therefore essential for slowing neurodegenerative progression (Subedi et al. 2019). Phytochemicals exert neuroprotective effects primarily through inhibition of NF‐κB–mediated inflammatory signaling and activation of Nrf2‐dependent antioxidant defense mechanisms (Subedi et al. 2019).

#### 6.4.1 NF‐κB Pathway and Suppression of Inflammation

By disrupting the pro‐inflammatory signaling cascade, certain phytochemicals act as molecular brakes on the brain's overactive immune response. Sulforaphane inhibits the nuclear translocation of nuclear factor kappa B, thereby suppressing the transcription of inflammatory genes (Seo et al. 2017). As a result, the production of pro‐inflammatory cytokines such as interleukin‐6 (IL‐6) and tumor necrosis factor‐α (TNF‐α) is markedly reduced (Seo et al. 2017). Andrographolide modulates the GSK‐3β/NF‐κB axis, leading to attenuation of microglial activation and robust suppression of neuroinflammatory responses (Yu, Shi, et al. 2024; Kehinde et al. 2025). This dual‐target mechanism contributes to the disruption of pathological crosstalk between inflammation, amyloid accumulation, and tau pathology (Kehinde et al. 2025). Curcumin further reinforces NF‐κB–centered anti‐inflammatory strategies by directly inhibiting NF‐κB activation and reducing glial reactivity in the Alzheimer's disease brain (Deng et al. 2014). Experimental studies demonstrate that curcumin downregulates the expression of pro‐inflammatory mediators, including IL‐1β, IL‐6, and TNF‐α, while suppressing astrocytic and microglial activation, thereby alleviating the chronic neuroinflammatory milieu associated with Aβ pathology (Deng et al. 2014; Tan et al. 2024). Expanding on these neuro‐immunomodulatory effects, Isoorientin reduces the number of activated microglia surrounding Aβ plaques, thereby dampening the local inflammatory microenvironment and mitigating neuroinflammatory burden (Feng et al. 2024).

#### 6.4.2 Nrf2/HO‐1 Pathway and Enhancement of Antioxidant Defense

Although the suppression of inflammation is vital the concurrent activation of the brain's internal defense systems is necessary to counteract the oxidative damage already present in the AD brain (Pathak and Kabra 2024). In this regard, Quercetin activates the nuclear factor erythroid 2–related factor 2 (Nrf2)–antioxidant response element (ARE) pathway, enhancing endogenous antioxidant defenses (Pathak and Kabra 2024). This activation increases the expression of antioxidant enzymes such as heme oxygenase‐1 (HO‐1) and superoxide dismutase (SOD), while reducing mitochondrial reactive oxygen species generation (Pathak and Kabra 2024). Complementing this antioxidant surge, Oxyphylla A enhances neuronal resistance to oxidative damage by activating the Nrf2–Keap1–HO‐1 signaling pathway (Bian et al. 2021). Through limiting lipid peroxidation and protein oxidation, this mechanism contributes to the preservation of neuronal integrity under oxidative stress conditions (Bian et al. 2021). Considering the complex and multifactorial pathogenesis of Alzheimer's disease, systematic screening of phytochemical libraries may provide a rational strategy for identifying compounds with multi‐target activity, a feature increasingly regarded as essential for effective therapeutic intervention in such complex disorders (Bian et al. 2021).

## 7 Comparative Perspective: Lecanemab and Donanemab vs. Phytochemicals

**FIGURE 4:**
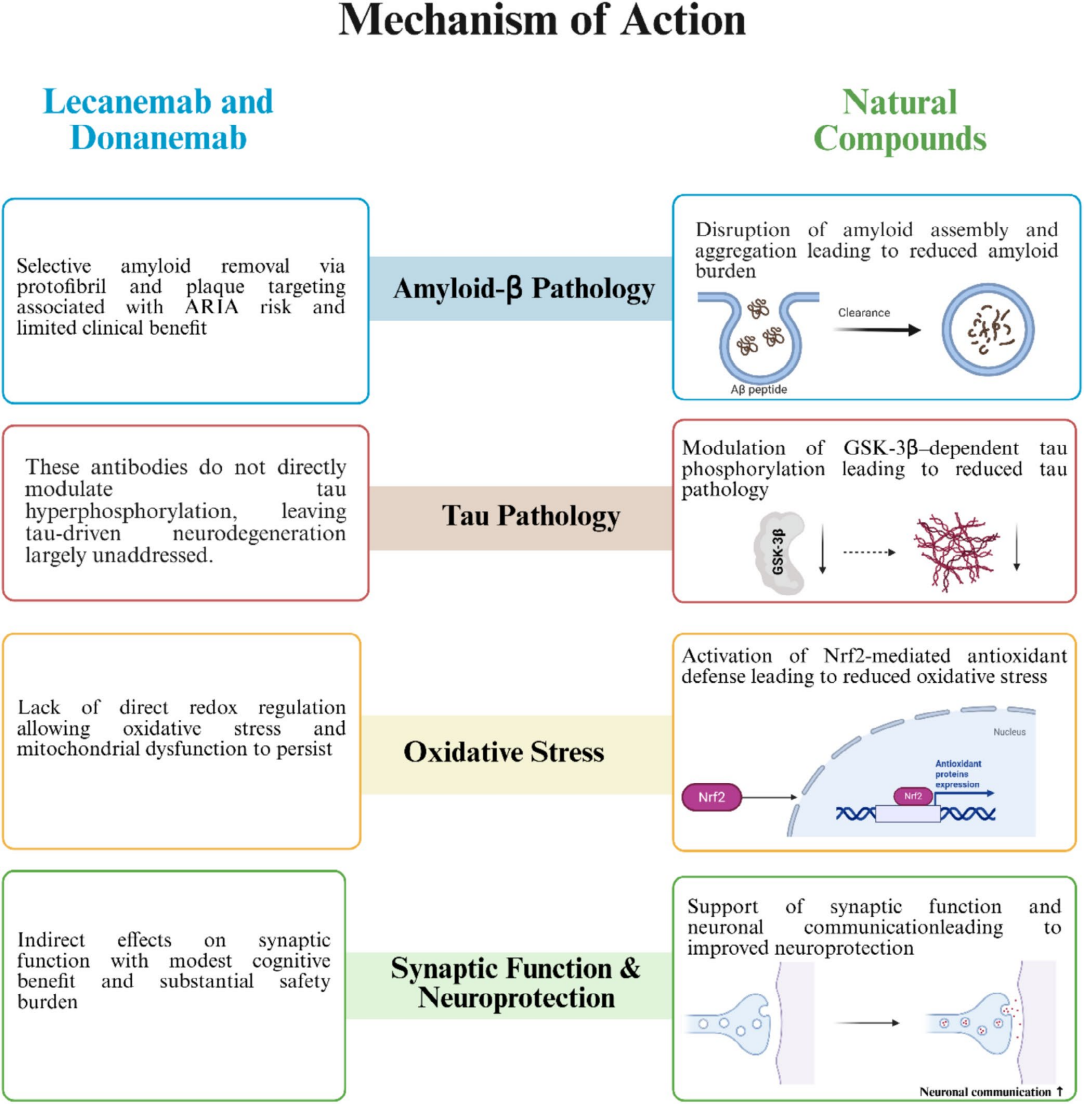
Comparative mechanisms of action of anti‐amyloid monoclonal antibodies and natural bioactive compounds in Alzheimer's disease.

Current drugs and disease‐modifying approaches have largely been designed to act on individual molecular targets, including cholinesterases, NMDA receptors, or amyloid‐β species, resulting in modest symptomatic benefits without durable disease modification (Cheong et al. 2022; Bui and Nguyen 2017). Even combination therapies suffer from pharmacokinetic constraints, drug–drug interactions, and limited long‐term efficacy, underscoring the structural limitations of single‐target paradigms in a systems‐level disease such as AD (Cheong et al. 2022). In contrast, accumulating evidence indicates that natural compounds represent promising alternatives or complements to conventional synthetic drugs, primarily due to their intrinsic multi‐target mechanisms of action (Angelova et al. 2024; Gong et al. 2022). Many phytochemicals simultaneously modulate multiple core pathological processes underlying AD, including amyloid‐β accumulation, tau hyperphosphorylation, oxidative stress, neuroinflammation, synaptic dysfunction, and mitochondrial impairment (Angelova et al. 2024; Xin et al. 2025) (Figure 4). This polypharmacological profile aligns more closely with the complex biology of AD and supports the rationale for multi‐target‐directed therapeutic strategies (Cheong et al. 2022; Xin et al. 2025).

A wide range of plant‐derived molecules, particularly flavonoids, alkaloids, terpenoids, and polyphenols, have demonstrated antioxidant, anti‐inflammatory, and neuroprotective properties that are critical for attenuating neurodegenerative cascades (Gong et al. 2022; Hossain and Hussain 2025). Several natural compounds, including curcumin, resveratrol, epigallocatechin‐3‐gallate (EGCG), myricetin, and grape seed proanthocyanidins, have been shown to directly interact with tau protein, inhibit tau aggregation, reduce tau hyperphosphorylation, and promote tau clearance through autophagic and proteasomal pathways in cellular and animal models (Gong et al. 2022; Shi and Zhao 2024). In addition, natural multi‐target agents such as the marine‐derived oligomannate GV‐971 illustrate how a single compound can concurrently influence amyloid aggregation, neuroinflammation, and gut–brain axis signaling (Xin et al. 2025). From a safety perspective, synthetic AD drugs frequently exhibit adverse effects that limit tolerability, particularly in elderly populations, contributing to treatment discontinuation and reduced adherence (Bui and Nguyen 2017; Kong et al. 2021). By contrast, phytochemicals are generally associated with more favorable safety profiles and broader therapeutic windows, partly reflecting their evolutionary optimization for biological compatibility (Kong et al. 2021; Islam et al. 2022). Importantly, several natural compounds also demonstrate effective blood–brain barrier permeability, a major limitation of many synthetic central nervous system drugs, thereby enhancing their translational potential for AD therapy (Hossain and Hussain 2025). These advantages aside, phytochemicals are not without challenges. Their clinical translation is constrained by limited bioavailability, variability in formulation and dosing, and the need for optimization of ADMET properties, including metabolic stability and brain penetration (Islam et al. 2022). Nevertheless, advances in medicinal chemistry, formulation strategies, and systems‐based drug design are increasingly enabling the refinement of natural compounds into viable multi‐target therapeutic candidates (Cheong et al. 2022; Xin et al. 2025). Viewed collectively, these observations highlight a fundamental contrast between single‐target synthetic drugs and multi‐target natural compounds, suggesting that phytochemicals may offer a more holistic and biologically congruent approach to addressing the complex pathophysiology of Alzheimer's disease, either as standalone agents or as adjuncts to existing therapies (Angelova et al. 2024; Yang, Zhou, et al. 2023) (Figure 4). Notwithstanding their conceptual advantages, phytochemicals are not without limitations. Their clinical applicability is hindered by poor bioavailability, variability in natural sources, and lack of standardized dosing protocols. In contrast, monoclonal antibodies provide well‐defined pharmacokinetic profiles but are limited by safety concerns and high cost. This contrast highlights a fundamental trade‐off between biological complexity and clinical practicality. Consequently, the future of Alzheimer's disease therapy may not lie in replacing one approach with another, but in integrating multi‐target phytochemical strategies with advanced drug delivery systems and precision medicine frameworks.

### 7.1 Challenges in Designing Multi‐Agent Clinical Trials

A significant but often overlooked obstacle in advancing polypharmacy strategies for AD is the methodological complexity of testing multiple co‐agents simultaneously in clinical trials (Cummings, Gold, et al. 2025). Traditional randomized controlled trials, primarily designed to evaluate single drugs, encounter major limitations with multi‐component treatments. These include the exponential increase in treatment arms required by full factorial designs (for instance, a 2 × 2 factorial testing an amyloid antibody combined with a phytochemical supplement would need four groups and large sample sizes to detect interaction effects), difficulties in attributing outcomes to individual components versus their interactions, and challenges in choosing co‐primary endpoints that reflect both amyloid reduction and broader neuroprotective or metabolic outcomes (Cummings, Burstein, and Fillit 2025). Other issues involve attributing safety signals when multiple agents are administered, managing pharmacokinetic interactions—especially when plant‐based compounds are combined with biologics or small drugs sharing metabolic pathways—and the regulatory challenges that are not well adapted to multi‐component therapies. Adaptive platform trials, such as those used in oncology (e.g., I‐SPY2, STAMPEDE), offer potential solutions by testing multiple co‐agents against a common control group and using response‐adaptive randomization (Angioni et al. 2025). Applying these designs to AD, where outcomes take 18–36 months to assess and placebo effects are difficult to control, presents a challenging yet feasible methodological frontier. The field must recognize these challenges rather than treat multi‐target therapies as a simple extension of existing trial frameworks (Angioni et al. 2025; Cummings, Gold, et al. 2025; Cummings, Burstein, and Fillit 2025).

### 7.2 The FINGER Model: Multi‐Domain Interventions in the Context of Amyloid Therapies

The Finnish Geriatric Intervention Study to Prevent Cognitive Impairment and Disability (FINGER) showed that a structured, multi‐domain lifestyle program—including dietary guidance based on the Nordic diet, aerobic and resistance exercises, cognitive training, and intensive management of cardiovascular and metabolic risks—significantly reduced cognitive decline over 2 years in older adults at risk of dementia (Lorenzon et al. 2025; Sakurai et al. 2025). This trial was groundbreaking as the first large‐scale RCT to demonstrate that a non‐drug, multi‐component intervention can mitigate cognitive decline in at‐risk individuals, confirming that AD's multifactorial nature can be tackled by targeting multiple modifiable risk factors simultaneously. Remarkably, this benefit was achieved without any amyloid‐targeting drugs, underscoring the role lifestyle factors play in enhancing cognitive reserve. The FINGER approach has inspired a global network of prevention trials, including the MAPT trial in France, the preDIVA study in the Netherlands, the MIND‐AD initiative, and the US POINTER trial—a large‐scale American adaptation of FINGER involving 2111 older adults at risk of dementia, which demonstrated that a structured multidomain lifestyle intervention (exercise, MIND diet, cognitive training, and cardiovascular monitoring) produced significantly greater improvement in global cognitive function compared to a lower‐intensity self‐guided approach over 2 years (Zwan et al. 2025; Sakurai et al. 2025; Baker et al. 2025). Despite varied outcomes, these studies collectively reinforce that no single intervention can effectively prevent or delay AD. Combining multiple strategies addressing pathological and lifestyle factors yields plausible, and potentially additive or synergistic, effects (Bereczki et al. 2025). In the emerging era of amyloid immunotherapy, FINGER provides a valuable model: it offers an established trial infrastructure that could be combined with pharmacological agents—such as disease‐modifying antibodies, phytochemicals, or repurposed drugs—to determine whether tackling both amyloid pathology and upstream risk factors leads to better clinical outcomes. Importantly, this combinatorial potential is not merely theoretical. Lecanemab and donanemab have demonstrated measurable reductions in cerebral amyloid burden, yet their clinical benefits remain modest—slowing cognitive decline by approximately 27% and 35% relative to placebo, respectively (van Dyck et al. 2023; Sims et al. 2023). This gap between robust target engagement and limited clinical impact strongly suggests that amyloid clearance alone is insufficient, and that residual pathological processes—including vascular dysfunction, metabolic dysregulation, neuroinflammation, and synaptic loss—continue to drive disease progression even after amyloid removal. A FINGER‐type multi‐domain intervention, applied concurrently with anti‐amyloid immunotherapy, could theoretically address precisely these residual mechanisms: dietary and exercise components would target vascular and metabolic co‐pathologies, cognitive training would support synaptic reserve, and cardiovascular risk monitoring would reduce cerebrovascular burden. In this sense, combining an anti‐amyloid antibody with a structured multi‐domain lifestyle program represents a biologically coherent and clinically testable strategy for maximizing therapeutic benefit—one that neither approach could achieve on its own (Cummings, Gold, et al. 2025; Bereczki et al. 2025; Sakurai et al. 2025). The MIND‐AD trial exemplifies this by evaluating a combined lifestyle intervention with vortioxetine in at‐risk individuals, illustrating how FINGER‐style designs can facilitate polypharmacy studies (Soldevila‐Domenech et al. 2025; Campagnoli et al. 2025). Similarly, the MET‐FINGER trial provides a particularly relevant example by combining the FINGER 2.0 multidomain lifestyle intervention with metformin repurposing in an APOE ε4‐enriched population, illustrating how lifestyle‐based multidomain frameworks can be integrated with pharmacological co‐agents in dementia prevention studies. Future insights into multi‐target AD treatments should explore integrating phytochemicals within this multi‐domain approach. Within such multidomain frameworks, phytochemicals may serve as biologically complementary co‐agents capable of simultaneously modulating oxidative stress, neuroinflammation, and metabolic dysfunction.

## 8 Conclusion and Future Directions

Alzheimer's disease is a highly complex and multifactorial neurodegenerative disorder driven by the interplay of amyloid‐β accumulation, tau pathology, neuroinflammation, oxidative stress, and synaptic dysfunction. The evidence presented in this review clearly demonstrates that these pathological processes do not act independently but rather form an interconnected network that collectively drives disease progression. This biological complexity significantly limits the effectiveness of conventional single‐target therapeutic approaches. Although recently developed anti‐amyloid monoclonal antibodies have demonstrated measurable reductions in amyloid burden, their modest clinical benefits, safety concerns, and high economic cost highlight the limitations of amyloid‐centric strategies. The observed disconnect between target engagement and meaningful clinical improvement challenges the long‐standing dominance of the amyloid cascade hypothesis as a sufficient therapeutic framework. In contrast, phytochemicals emerge as promising multi‐target agents capable of simultaneously modulating multiple disease‐relevant pathways. Their ability to influence amyloid processing, tau phosphorylation, neuroinflammation, oxidative stress, and synaptic integrity positions them as biologically aligned candidates for addressing the systemic nature of Alzheimer's disease. However, it is important to acknowledge that the current body of evidence supporting phytochemical‐based therapies is largely derived from in vitro and preclinical studies, with limited validation in large‐scale clinical trials. This lack of clinical translation represents a major bottleneck that must be addressed before these compounds can be considered viable therapeutic alternatives. Despite these challenges, advances in drug delivery systems, nanotechnology, and medicinal chemistry provide new opportunities to enhance the bioavailability, stability, and brain penetration of phytochemicals. In addition, emerging strategies such as multi‐target drug design and PROTAC‐based targeted protein degradation may further expand their therapeutic potential. The integration of phytochemicals with these modern approaches could enable more precise and effective modulation of the complex pathological networks underlying Alzheimer's disease. In this context, the present review contributes to the field by providing an integrated, systems‐level perspective that connects amyloid pathology, tau dysregulation, neuroinflammation, oxidative stress, and synaptic dysfunction within a unified multi‐target therapeutic framework. This perspective not only highlights the limitations of current single‐target strategies but also underscores the potential of phytochemical‐based interventions as part of next‐generation therapeutic approaches. Ultimately, future progress in Alzheimer's disease therapy will depend on moving beyond reductionist paradigms toward comprehensive, multi‐target strategies that reflect the true biological complexity of the disease. Future therapeutic progress in AD will likely require not only the optimization of individual phytochemicals but also their rational integration into broader polypharmacological and multidomain intervention frameworks, including lifestyle‐based strategies, repurposed drugs, and amyloid‐targeting therapies.

## Author Contributions

**İlay Yurt:** writing – original draft, methodology, visualization. **Yağmur Nisa Cerlet:** methodology, writing – original draft. **Haşmet Ayhan Hanağası:** supervision, writing – review and editing, writing – original draft. **Emre Aktaş:** conceptualization, writing – original draft, visualization, methodology.

## Funding

The authors have nothing to report.

## Ethics Statement

The authors have nothing to report.

## Conflicts of Interest

The authors declare no conflicts of interest.

## Data Availability

The authors have nothing to report.
